# Tuning Aerosol Particle Size Distribution of Metered Dose Inhalers Using Cosolvents and Surfactants

**DOI:** 10.1155/2013/574310

**Published:** 2013-07-24

**Authors:** Imran Y. Saleem, Hugh D. C. Smyth

**Affiliations:** ^1^School of Pharmacy & Biomolecular Sciences, Liverpool John Moores University, Liverpool L3 3AF, UK; ^2^Division of Pharmaceutics, College of Pharmacy, University of Texas at Austin, Austin, TX 78712-1113, USA

## Abstract

*Objectives.* The purpose of these studies was to understand the influence of cosolvent and surfactant contributions to particle size distributions emitted from solution metered dose inhalers (pMDIs) based on the propellant HFA 227. *Methods.* Two sets of formulations were prepared: (a) pMDIs-HFA 227 containing cosolvent (5–15% w/w ethanol) with constant surfactant (pluronic) concentration and (b) pMDIs-HFA 227 containing surfactant (0–5.45% w/w pluronic) with constant cosolvent concentration. Particle size distributions emitted from these pMDIs were analyzed using aerodynamic characterization (inertial impaction) and laser diffraction methods. *Results*. Both cosolvent and surfactant concentrations were positively correlated with median particle sizes; that is, drug particle size increased with increasing ethanol and pluronic concentrations. However, evaluation of particle size distributions showed that cosolvent caused reduction in the fine particle mode magnitude while the surfactant caused a shift in the mode position. These findings highlight the different mechanisms by which these components influence droplet formation and demonstrate the ability to utilize the different effects in formulations of pMDI-HFA 227 for independently modulating particle sizes in the respirable region. *Conclusion*. Potentially, the formulation design window generated using these excipients in combination could be used to match the particle size output of reformulated products to preexisting pMDI products.

## 1. Introduction

Pressurized metered dose inhalers (pMDIs) have found frequent application in the delivery of therapeutics for treatment of pulmonary diseases, such as asthma and chronic obstructive pulmonary disease [1]. They are compact and have the potential to deliver a wide range of doses and drugs [2]. In general, pMDI formulations comprise a therapeutic material, a propellant, cosolvents, and surfactants toprevent drug-aerosol coagulation and lubricate moving parts of the metering valve. 

Historically, pMDIs propellants were ozone-depleting chlorofluorocarbons (CFCs) [3]. This prompted the well-documented search for more environmentally friendly alternatives, such as the biocompatible, nonozone depleting hydrofluoroalkanes (tetrafluoroethane [HFA-134] and heptafluoropropane [HFA 227]) [4, 5]. Many of the presently marketed pMDIs contain HFA-134, as it was the first propellant to have full term toxicity testing [6]. However, despite some similarities in densities and vapor pressures, the physicochemical properties of HFAs and CFCs differ significantly [7, 8], leading to incompatibilities with traditional excipients, canister components, and valve elastomers. This has led to difficulties for reformulators trying to match the performance of newer generation formulations to those they are attempting to replace. The particle size distribution of inhalation aerosols is a critical parameter that needs to be carefully controlled since it determines where the aerosol will deposit in the respiratory tract and is closely linked to the efficacy as well as the side effects of the delivered medication as evidenced by the CFC to HFA transition [9, 10]. Furthermore, the particle size distribution of a pMDI aerosol depends on the physicochemical properties of the formulation [11]. Thus, reformulating pMDIs from CFCs to HFAs has been problematic, particularly when attempts have been made to match the performance of these two systems (aerosol output equivalence). This approach of reformulating pMDIs based on bioequivalency to the CFC pMDIs they have replaced was adopted by most companies involved in the transition from CFC to HFAs [8]. To achieve equivalence, there are two general strategies which can be used to modulate aerosol output and lung deposition of pMDIs; the first is altering the formulation parameters such as propellant and excipients which we have previously reviewed [8, 11]. Secondly, device design can be changed rationally to induce changes in aerosol output as we have previously shown for actuator nozzle dimensions [12, 13].

Part of the issue for matching deposition profiles of different products has been the difficulty in matching aerosol output beyond the mass median aerodynamic diameters and other composite measures such as fine particle fraction or respirable fractions. Indeed, it has been reported that HFA-134 solution formulations displayed multimodal particle size distributions. In these studies, varying cosolvent concentrations in HFA-134/ethanol pMDIs influenced particle size distribution but did not cause particle size modes to shift [5, 11]. This is problematic for formulators wishing to show equivalence with monomodal aerosols since changes in particle size output modulated by use of cosolvents alone will be insufficient to obtain a match of aerosol deposition profiles. 

In the current studies, we show that by controlling both cosolvent and surfactant concentrations, the aerosol particle size distributions can be modulated both along the *x* and *y* axes; that is, HFA 227 solution formulations can be tuned within a performance space. Specifically, the aim of these studies was to investigate the influence of changing the concentrations of a model surfactant (Pluronic L81) and ethanol on the emitted particle size distribution and *in vitro* aerosol deposition studies.

## 2. Materials and Methods

### 2.1. Materials

The propellant 1,1,1,2,3,3,3-heptafluoropropane (HFA 227) was provided as a gift from Solvay Fluorides Inc. (Houston, TX, USA). Fluorescein sodium (fluorescein Na) was purchased from Sigma-Aldrich (St. Louis, MO, USA). Ethanol (EtOH) (HPLC grade) and methanol (HPLC grade) were purchased from VWR (West Chester, PA, USA). Aluminium aerosol canisters and metered dose valves (25 *μ*L) were provided as a gift from Valois Pharmaceuticals (Marly-le-Roi, France). Pluronic L81 was a gift from BASF Corp (Parsippany, NJ, USA).

### 2.2. Preparation of Pressurised Metered Dose Inhalers (pMDIs)

A series of formulations were prepared (Table 1) containing various amounts by weight of Pluronic L81 and HFA 227 propellant with constant ethanol concentration (% w/w) and fluorescein Na drug mass (% w/w), as a model drug.  Table 2 represents formulations consisting of different ethanol concentrations (% w/w) but maintaining constant Pluronic L81 (% w/w) and fluorescein Na (% w/w) concentrations. Significant preformulation, solubility, and stability studies were performed to ensure compatibility of the model drug, excipients, and propellants (data not shown). 

Aliquots of Pluronic L81, ethanol, and fluorescein Na, as represented in Tables 1 and 2, were added by weight to aluminium aerosol canisters. Metered dose valves were then crimped onto the canisters using a manual canister crimper (Aero-Tech Laboratory Equipment Company, Worcester, NY) and filled with the desired weight of HFA 227 propellant using a pressure burette (Aero-Tech Laboratory Equipment Company, Worcester, NY, USA). All canisters were equipped with a 0.33 *μ*m actuator (Valois Pharmaceuticals, Marly-le-Roi, France) and prepared on the same day as testing.

### 2.3. Particle Size Analysis of Drug (Fluorescein Na) Using Laser Diffraction

Particle size characteristics of each of the formulations were determined using a Sympatec Helos laser diffraction instrument (Sympatec GmbH, Germany). The pMDI was positioned at a set distance from the laser beam and at a fixed height ensuring that the aerosol plume was projected across the laser. This was achieved by placing the aerosol device such that the actuator orifice was within the lens cut-off distance and was aligned with the height of the laser path. Particle size distribution was measured using four actuations shaking the canister between actuations five times and expressed as the volume median diameter (VMD) (*n* = 4).

### 2.4. Aerodynamic Particle Size Distributions of Drug (Fluorescein Na) Using the Next Generation Impactor (NGI)

Aerodynamic particle size distributions were determined from cascade impaction studies using a Next Generation Impactor (NGI) (MSP Corp, MN, USA) containing a United States Pharmacopeia induction port and operated at a flow rate of 30 L/min, precalibrated using a Gilmont Flowmeter Base Model F-4001 (Barnant Company, Barrington, IL, USA). The metering valves were primed by discharging three shots to waste. The pump was switched on 5 seconds prior to pMDI discharge, and pMDI was actuated ten times (*n* = 3). The emitted dose (ED) was expressed as the total mass of drug emitted from the inhaler. The fine particle fraction (% FPF) (defined as the mass of drug deposited (*d*_ae_ ≤ 4.6 *μ*m) was expressed as a percentage of the emitted dose and the fine particle dose (FPD) was expressed as the mass of drug deposited in the NGI (*d*_ae_ ≤ 4.6 *μ*m). Furthermore, inertial impaction data was also subjected to log-probability analysis to allow the derivation of mass median aerodynamic diameter (MMAD) and geometric standard deviation (GSD) for each formulation [14]. 

### 2.5. Chemical Analysis

Fluorescein Na captured on the actuator, induction port, and stages was extracted with methanol (HPLC grade). The aerodynamic particle size distribution was determined by analyzing each of the collected samples for fluorescein Na content by HPLC using a Hitachi Elite LaChrom (Hitachi, CA, USA) with UV detection at 490 nm using a Kromasil C8 column (150 mm × 4.6 mm i.d., Column Engineering, CA, USA). The mobile phase consisted of methanol : water (60 : 40) at a flow rate of 1.0 mL/min, injection volume 10 *μ*L, and quantification was by peak area using a standard curve in the range 0–25 *μ*g/mL.

### 2.6. Statistical Analysis

The formulations were compared with each other by means of a one-way ANOVA with Tukey's comparison test. The statistical significance level was set at *P* ≤ 0.05. 

## 3. Results

### 3.1. Effect of Surfactant Concentration on Particle Size Distributions Emitted from HFA 227 pMDIs

Investigations looked at the influence of altering surfactant levels in the HFA 227 pMDIs on aerosolization and particle size. Laser diffraction data (Table 3) illustrated a direct correlation between increasing surfactant concentration resulting in larger particle size (VMD). 

**Figure 1:**
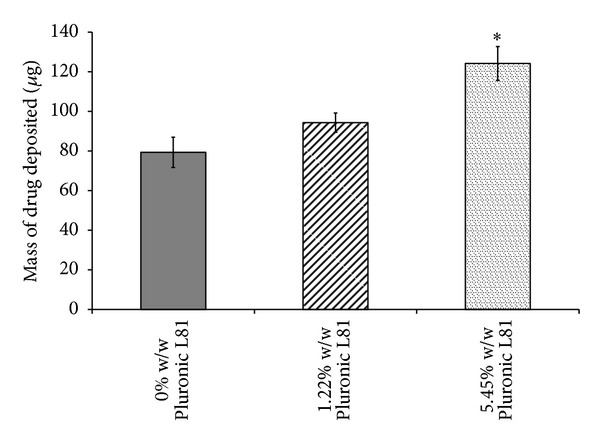
Comparison of HFA 227 formulations with varying concentrations of Pluronic L81 in terms of drug deposited in USP throat of NGI maintaining constant concentration of fluorescein Na and ethanol (data represent mean ± SD, *n* = 3). *P < 0.05 (ANOVA/Tukey's) 5.45% w/w Pluronic L81 versus 1.22 & 0% w/w Pluronic L81.

**Figure 2:**
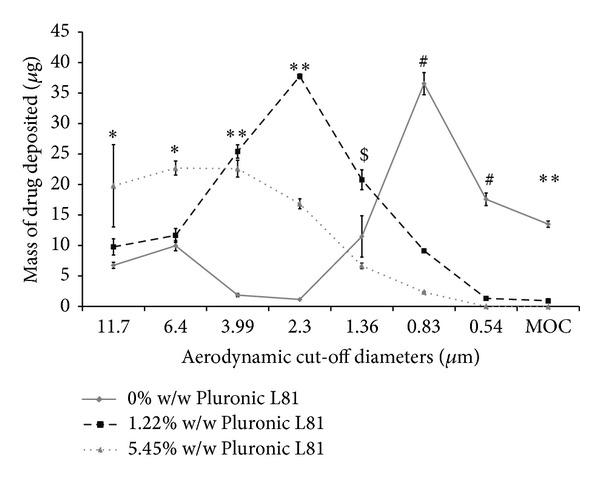
Comparison of HFA227 formulations in terms of drug deposited on each stage of Next Generation Impactor, maintaining constant concentration of ethanol (data represent mean ± SD, *n* = 3). MOC: microorifice collector. *P < 0.05 (ANOVA/Tukey's) 5.45% w/w Pluronic L81 versus 0 and 1.22% w/w Pluronic L81, **P < 0.05 (ANOVA/Tukey's) comparing all formulations, ^$^ *P* < 0.05 (ANOVA/Tukey's) 1% w/w Pluronic L81 versus 0 and 5% w/w Pluronic L81, and ^#^ *P* < 0.05 (ANOVA/Tukey's) 0% w/w Pluronic L81 versus 1.22 and 5% w/w Pluronic L81.

This was further reflected in the MMAD and emitted dose data from cascade impaction studies. However, a significant decrease in FPD and FPF was observed as the surfactant concentration was increased from 1.22% w/w to 5.45% w/w (*P* < 0.05, ANOVA/Tukey's). Similarly, USP induction port deposition was positively correlated with surfactant concentrations, with 5.45% w/w Pluronic L81 formulations resulting in significantly greater throat deposition compared to the other formulations (*P* < 0.05, ANOVA/Tukey's) (see Figure 1). In addition, increasing the concentration of surfactants caused the fine particle mode (population of particles less than ~10 microns) to shift along the abscissa. High surfactant concentrations (5.45% w/w) significantly dominated at cut-off diameters ≥6.4 *μ*m (*P* < 0.05, ANOVA/Tukey's), with 1% w/w Pluronic L81 dominating between 3.99–1.36 *μ*m (*P* < 0.05, ANOVA/Tukey's) (see Figure 2). 

### 3.2. Effect of Ethanol Concentration on Particle Size Distributions Emitted from HFA 227 pMDIs

A summary of the studies for fluorescein Na labelled pMDI formulations with varying levels of cosolvent is presented in Table 4. There was a direct correlation between increasing ethanol concentration resulting in enhanced particle size (VMD) and MMAD, with significant differences noted between formulations containing 15% w/w versus 10 or 5% w/w ethanol (*P* < 0.05, ANOVA/Tukey's).

**Figure 3:**
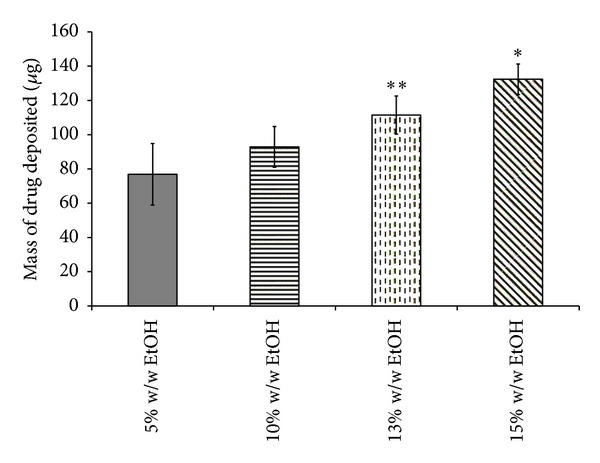
Comparison of HFA 227 formulations in terms of drug deposited in USP throat of NGI maintaining constant concentration of fluorescein Na and Pluronic L81 (data represent mean ± SD, *n* = 3). *P < 0.05 (ANOVA/Tukey's) 15% w/w EtOH versus 10 & 5% w/w EtOH, **P < 0.05 (ANOVA/Tukey's) 13% w/w EtOH versus 5% w/w EtOH.

**Figure 4:**
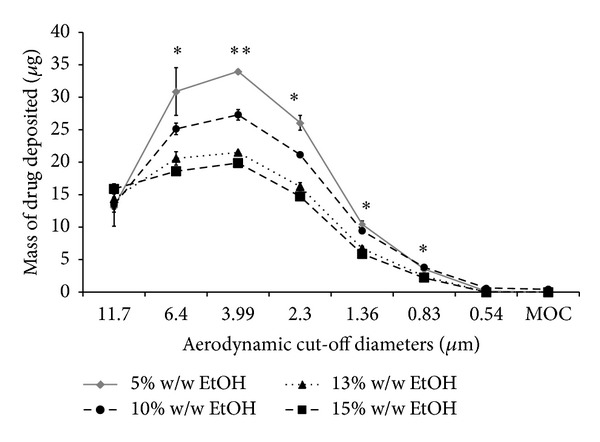
The influence of ethanol cosolvent concentrations on the aerodynamic particle size characteristics of HFA 227 formulations as quantified using a Next Generation Impactor. Surfactant concentrations were kept constant (data represent mean ± SD, *n* = 3). MOC: microorifice collector. *P < 0.05 (ANOVA/Tukey's) 15% w/w EtOH versus 10 and 5% w/w EtOH and **P < 0.05 comparing all formulations.

Although a decrease was noted in MMAD between formulations containing 5 and 10% w/w EtOH, this was not significant (*P* > 0.05, ANOVA/Tukey's). Furthermore, as MMAD values increased, this corresponds to a significantly decreased FPD between all formulations from 105.07 ± 2.31 *μ*g to 61.28 ± 1.79 *μ*g (Table 4) (*P* < 0.05, ANOVA/Tukey's). In addition, a decrease in FPF was also noted with significant difference comparing formulations with 15 and 13% w/w versus 10 and 5% w/w EtOH (*P* < 0.05, ANOVA/Tukey's). However, there was no significant difference in emitted doses between the formulations (*P* > 0.05, ANOVA/Tukey's). These observations are not surprising and are consistent with our previous studies conducted using HFA 134a propellants [11]. Thus, as expected with increased particle size, the USP induction port (throat) deposition also increased with increasing ethanol concentrations (see Figure 3). Furthermore, increasing ethanol concentrations in the HFA 227 formulations caused similar changes to the particle size distributions (see Figure 4). Generally, the lower ethanol concentrations (5 and 10% w/w), the greater the mass of drug deposited at cut-off-diameters 6.4 to 0.83 *μ*m (*P* < 0.05, ANOVA/Tukey's). Importantly, the positions of the particle size modes were not changed as ethanol concentrations are changed, rather their magnitudes were altered. These observations are in contrast to those discussed previously when we varied the surfactant concentration while keeping ethanol concentrations constant. 

## 4. Discussion

This study investigated the effects of ethanol and surfactant (Pluronic L81) concentrations on drug particle size distribution and *in vitro* drug aerosol deposition using pMDIs containing propellant HFA 227. For solution-based pMDIs, these two excipient classes are common and often necessary for ensuring solubility and performance. Some previous studies have developed predictive models for solution-based formulations [15], but in general, these approaches have been to predict MMAD or fine particle fractions. Due to the dependency of pharmacokinetics and pharmacodynamics of inhaled products on deposition patterns, particularly in the challenging cases of matching performance, it may be more useful to match whole distributions rather than measures of central tendency or respirability. 

Although pluronics are not currently used in marketed pMDI products, several recent patents and publications have detailed the use of these surfactants in this manner [16–19]. The main motivation for this work was to determine if the *in vitro *aerosol performance of these model systems could be adequately modulated using the two components mentioned previously. Several literature reports confirm that increasing either cosolvent or surfactant concentrations resulted in increased emitted particle sizes. We observed that ethanol and the Pluronic L81 surfactant caused very different effects on the emitted particle size distributions when their concentrations were altered. This was significant for several reasons. Firstly, it appears that the mechanisms by which droplet sizes were influenced by cosolvent versus surfactants were very different. Differing mechanisms would indicate that particle size could be manipulated independently using these two approaches. Furthermore, modulating drug particle size is important for either optimizing the performance of these products or matching the performance of products to those already approved by regulatory agencies across the world. Secondly, the pathway to regulatory approval of products (generic or otherwise) appears to focus on stage-by-stage deposition rather than mean or median values that are less sensitive markers of deposition. Therefore, the focus of many studies and literature reports has been MMAD and FPD and the ability to manipulate particle size distribution for lung targeting. This is considered an important aspect of formulation as the Food and Drug Administration (FDA) and other regulatory agencies are interested in cascade impactor stage-by-stage *in vitro* correlation. In this study, consistent with several previous investigations, we showed that varying concentrations of cosolvent (ethanol) and surfactant (Pluronic L81) caused differences in aerosol particle sizes.

Perhaps the most challenging aspect in reformulating pMDIs, with surfactants traditionally employed in CFC-based pMDIs and found in FDA-approved products, is the limited solubility in the more polar HFAs [7, 20]. Surfactants are generally required in solution and dispersion formulations as solubilising/dispersing agents and as valve lubricants [5]. Cosolvents are generally required in HFA-based formulations to aid in the solubilisation of surfactants [5] which could affect the vapor pressure of pMDI mixtures and thus the aerosol respirable fraction. The development of novel surfactants for HFA-based pMDIs has been limited but several groups focusing on this task using suspension based pMDIs [21], and recently pluronic copolymers have been investigated as potential surfactants in solution based pMDI formulations by Ridder et al. [22] who used Pluronic L81 surfactant and found good solubility in HFA 227.

As Pluronic L81 concentrations were increased, a population modal shift to higher particle sizes was observed. The elevated MMAD and VMD values associated with higher concentrations of Pluronic L81 may be attributed to strong hydrogen bonding between Pluronic L81 and HFA 227 and the surface active nature of Pluronic L81 resulting in decreased evaporation rates from droplet surfaces [8, 23]. Similar to cosolvent effects, another explanation is the decreased propellant fraction leading to reduced vapor pressures and reduction of atomization energy at the nozzle [8]. Consequently, the increase in MMAD and VMD with increasing Pluronic L81 concentration from 1.22 to 5.45% w/w resulted in a greater deposition of drug in the throat, reduced the fraction of emitted dose with *d*_ae_ ≤ 4.6 *μ*m, and hence reduced FPF and FPD. However, there appear to be important differences in the mechanism of particle size modulation between ethanol and Pluronic L81 (see Figures 2 and 4). It can be seen that the effects of increasing surfactant concentration are somewhat different from those patterns observed with cosolvents which had the effect of moving the particle size distributions along the ordinate axis and not the abscissa.

Considerable work on solution formulations has been reported by several groups including Stein et al. from 3 M [24–28]. The correlation of increasing cosolvent concentrations resulting in enhanced particle sizes has been attributed to the reduced energy available for atomization due to the decreased vapor pressures [11] and increased droplet sizes owing to either slow or incomplete evaporation [29] at these time scales. These studies show that ethanol caused a decrease in the relative proportion of fine particles due to the decrease in vapor pressure of the solution as we add ethanol. The amount of fine particles in the aerosol cloud is directly proportional to the square root of the pressure—as ethanol concentration increases, the vapor pressure of the solution decreases; hence, the number of fine particles also decreases. This was supported by the observation of greater drug deposition in the USP induction port for formulations of higher ethanol content in our investigations. Moreover, the laser diffraction data (obtained before significant evaporation could occur) appears to support this theory, showing much higher particle sizes than those obtained from cascade impaction studies [26, 30, 31].

**Figure 5:**
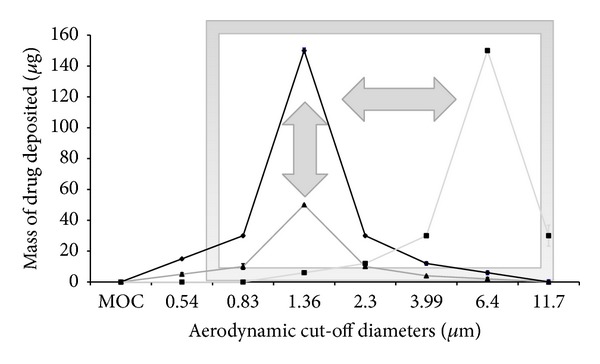
Concept of the design window illustrated for solution-based HFAs containing cosolvent (changing amplitude of the particle size mode, vertical tuning) and surfactants (changing location of the mode, horizontal tuning) in which particle size modulation may be achieved by manipulation of these two components that act through independent mechanisms to effect particle size change.

With these differences in mind, we hypothesize that the particle size “fingerprint” for HFA solution formulations may be unique to the excipient selection and relative concentrations. If the mechanisms by which particle size distributions are modulated by cosolvents and surfactants are independent of each other, a design space may be generated for each system (see Figure 5). In terms of reformulation efforts and development of equivalent generic pMDIs, this type of approach could be used to match the stage-by-stage analysis or particle size fingerprint more rapidly. Studies in our labs are currently underway to thoroughly test these hypotheses using surface response analysis.

## 5. Conclusion

The purpose of these studies was to understand cosolvent and surfactant contributions to particle size distributions emitted from solution metered dose inhalers based on the propellant HFA 227. These studies build on several previous published investigations using the propellant HFA 134a. Here, for the first time, we describe how particle size distributions can be modulated differently using two different formulation excipients by shifting size distribution modes to different locations and by modifying the amplitude of the modes. The practical implications of using these excipients to independently modulate particle size distributions are that a formulation window can be generated from which reformulation or bioequivalence research and development can be facilitated.

## Figures and Tables

**Table 1:** Composition of HFA 227 formulations maintaining constant fluorescein Na and ethanol concentration (*n* = 3).

Formulation	Pluronic L81 (% w/w)	Ethanol (% w/w)	Fluorescein Na (% w/w)
1.22% w/w Pluronic L81	1.22	13.3	0.04
5.45% w/w Pluronic L81	5.45	13.4	0.04
0% w/w Pluronic L81	0*	13.3	0.04

*Formulation 0% w/w Pluronic L81 contained 1.22% w/w of deionized water as a replacement for Pluronic L81 to maintain mass balance.

**Table 2:** Composition of HFA 227 formulations maintaining constant Pluronic L81 and fluorescein Na concentrations (*n* = 3).

Formulation	Pluronic L81 (% w/w)	Ethanol (% w/w)	Fluorescein Na (% w/w)
5% w/w EtOH	5	5	0.04
10% w/w EtOH	5	10	0.04
13% w/w EtOH	5	13	0.04
15% w/w EtOH	5	15	0.04

**Table 3:** Comparison of formulations with different concentrations of Pluronic L81 (data represent mean ± SD, *n* = 3).

	Pluronic L81 Concentration (% w/w)		
	0	1.22	5.45
ED (*μ*g)	170.69 ± 21.85**	211.14 ± 9.63	215.14 ± 11.55
FPF (%)	49.63 ± 2.00	50.72 ± 0.69	33.20 ± 3.37*
FPD (*μ*g)	84.61 ± 10.19	107.05 ± 3.53	71.17 ± 3.79^#^
MMAD (*μ*m)	1.56 ± 0.05^$^	3.70 ± 0.08^$^	5.93 ± 0.49^$^
GSD	3.22 ± 0.31	2.00 ± 0.05	1.78 ± 0.07
VMD (*μ*m)	7.07 ± 0.14	8.39 ± 0.11	11.04 ± 0.46*

ED: emitted dose, MMAD: mass median aerodynamic diameter, FPF: fine particle fraction, FPD: fine particle dose, and GSD: geometric standard deviation. **P < 0.05 (ANOVA/Tukey's) 0% w/w Pluronic L81 versus 1.22 and 5.45% w/w Pluronic L81, *P < 0.05 (ANOVA/Tukey's) 5.45% w/w Pluronic L81 versus 0 and 1.22% w/w Pluronic L81, ^#^ *P* < 0.05 (ANOVA/Tukey's) 5.45% w/w Pluronic L81 versus 1.22% w/w Pluronic L81, and ^$^ *P* < 0.05 comparing all formulations.

**Table 4:** Comparison of HFA 227 formulations with different concentrations of ethanol (data represent mean ± SD, *n* = 3).

	Ethanol concentration (% w/w)/(vapor pressure, psi)			
	5 (59.8 psi)	10 (54.6 psi)	13 (52.8 psi)	15 (50.2 psi)
ED (*μ*g)	195.08 ± 23.25	194.26 ± 14.19	193.31 ± 12.44	209.52 ± 11.02
FPF (%)	54.31 ± 5.65	45.39 ± 2.90	35.02 ± 2.26**	29.27 ± 0.77*
FPD (*μ*g)	105.07 ± 2.31^±^	87.91 ± 1.73^±^	67.52 ± 0.38^±^	61.28 ± 1.79^±^
MMAD (*μ*m)	5.13 ± 0.23	5.05 ± 0.15	5.55 ± 0.18	5.79 ± 0.05*
GSD	1.74 ± 0.12	2.07 ± 0.01	1.73 ± 0.03	1.78 ± 0.01
VMD (*μ*m)	5.32 ± 0.54	8.03 ± 0.36	9.72 ± 0.56	12.0 ± 0.44*

ED: emitted dose, MMAD: mass median aerodynamic diameter, FPF: fine particle fraction, FPD: fine particle dose, and GSD: geometric standard deviation. *P < 0.05 (ANOVA/Tukey's) 15% w/w EtOH versus 10 and 5% w/w EtOH, **P < 0.05 (ANOVA/Tukey's) 13% w/w EtOH versus 10 and 5% w/w EtOH, and ^±^ *P* < 0.05 comparing all formulations (ANOVA/Tukey's).
